# Antioxidant and Cytotoxic Activities of *Centella asiatica* (L) Urb.

**DOI:** 10.3390/ijms10093713

**Published:** 2009-08-26

**Authors:** Frederico Pittella, Rafael C. Dutra, Dalton D. Junior, Miriam T. P. Lopes, Nádia R. Barbosa

**Affiliations:** 1 Departamento de Alimentos e Toxicologia, Faculdade de Farmácia e Bioquímica, Universidade Federal de Juiz de Fora, Martelos, CEP 36036-330, Juiz de Fora, MG, Brazil; E-Mail:fredpittella@gmail.com (F.P.); 2 Laboratório de Substâncias Antitumorais, Instituto de Ciências Biológicas, Universidade Federal de Minas Gerais, CEP 31270-901, Belo Horizonte, MG, Brazil; E-Mail:mtpl@icb.ufmg.br (M.T.P.L.)

**Keywords:** Centella asiatica, Umbelliferae, phenolic constituents, DPPH, cytotoxic

## Abstract

In the present study, the phenolic (Folin-Dennis) and flavonoid (colorimetric assay) constituents, antioxidant [2,2-diphenyl-2-picrylhydrazyl hydrate (DPPH) assay] and cytotoxic activities of an aqueous extract (AE) of *Centella asiatica* leaves were investigated. The aqueous extract (50 g/L) was obtained by infusion followed by cold maceration for 24 h. The levels of phenolic and flavonoid compounds were 2.86 g/100 g and 0.361 g/100 g, respectively. The AE showed elevated DPPH scavenging activity, with an IC_50_ value of 31.25 μg/mL. The AE had a promising activity against mouse melanoma (B_16_F_1_), human breast cancer (MDA MB-231) and rat glioma (C_6_) cell lines, with IC_50_ values of 698.0, 648.0 and 1000.0 μg/mL, respectively. A positive correlation was established between the level of flavonoids, antioxidant and antitumor activities.

## 1. Introduction

Aging is a natural process which is related to several morphological and biochemical changes that happens from maturity to senescence, making the organism vulnerable to diseases and toxicity, and eventually leading to cellular death. According to the hypothesis of oxidative stress on aging, the loss of functional capacity associated to senescence comes from accumulation of molecular oxidative damages [1] brought about by (toxic) free radicals produced during normal breathing. Free radicals were previously reported as being capable of damaging a lot of cellular components such as proteins, lipids and DNA [2].

To protect the cells from oxidative damages by oxidants, produced during oxygen methabolism, an antioxidant system is used by aerobic organisms. The main antioxidant agents such as superoxide dismutase (SOD), catalase, glutathione peroxidase (GSH-Px), glutathione, ascorbic acid and tocopherol are important for cellular protection, due to their ability to eliminate free radicals, such as reactive oxygen species (ROS) [3]. Nowadays, there is an increasing interest in the biochemical functions of natural antioxidant extracts from vegetables, fruits, and medicinal plants, which can become candidates to prevent oxidative damage, promoting health. The phenolic constituents found in vegetables have received considerable attention for being the main components of antioxidant activity, in spite of not being the only ones. The antioxidant activity of phenolic constituents has been attributed to its oxide-reduction properties, which play an important role in the adsorption or neutralization of free radicals [4].

Medicinal plants have been a useful source for the research of new biologically active compounds. Different approaches are used to select a plant for research, specially the ethno-medical data approach. Unfortunately, the ethno-medical data is not always completely reliable, since it is difficult to diagnosed cancer well. Apart from the medicinal effects of traditional herbs, exploratory researches have been made and a wide variety of new biological activities from traditional medicinal plants have recently been reported, including anticancer activity [5].

*Centella asiatica* (L) Urb., popularly known in Brazil as cairuçu-asiático, centelha, codagem and pata-de-mula [6], is a cosmopolitan member of the Umbelliferae family that presents pantropical distribution. It is a perennial herb that has been used for centuries in Ayurvedic medicine to treat several disorders, such as insanity, asthma, leprosy, ulcers and eczema and for wound healing [7,8]. *Centella asiatica* contains triterpene glycosides such as centellasaponin, asiaticoside, madecassoside and sceffoleoside [9], and also asiatic acid and madecassic acid [10,11]. Asiaticoside is the most abundant triterpene glycoside in the water extract and it is transformed into asiatic acid *in vivo* by hydrolysis. Although the asiatic acid has shown cytotoxic activity on fibroblast cells [12] and induces apoptosis in different sorts of cancer [13–18], to date no scientific report related the presence of phenolic compounds of *Centella asiatica* to cytotoxic activity. Consequently, we have focused on establishing a relationship between the total phenolic content and antioxidant activity with cytotoxic activity, evaluating the activity against cancer cell lines using aqueous extract (AE) obtained from *Centella asiatica* leaves.

## 2. Results and Discussion

The AE showed a phenolic constituents level of 2.86 g/100 g. A recent report [19] showed that the method used quantifies mainly high-molecular weight tannins, such as hydrolysable tannin and other polyphenols that have a molecule of gallic acid in its structure, absorbing energy at the wavelength of 760 nm. In fact, values found in this study are in agreement with the results published recently which obtained values ranging from 3.23–11.7 g/100 g for different parts of *Centella asiatica* [20].

The flavonoids level in the AE was 0.361 g/100 g. Flavonoids are highly polar molecules of low molecular weight which absorb energy around 420 nm. Water is a polar extractant, so it will extract polar constituents, such as heterosides. In this work, water was used as liquid extractant and the level of flavonoids observed was high, suggesting that these flavonoid constituents might be in the heteroside form, since previous studies showed that flavonoids can be found in nature in the free state or in the form of glycosides [21,22].

The antioxidant activity of AE of *Centella asiatica* was evaluated by its ability to scavenge DPPH free radicals. The radical scavenging activity of the compounds can be measured by the decolorizing effect following the trapping of the unpaired electrons of DPPH. The AE showed a high antioxidant activity, with an IC_50_ value of 31.25 μg/mL. Ascorbic acid and butylated hydroxytoluene (BHT) produced IC_50_ values of 2.50 μg/mL and 7.58 μg/mL, respectively. Based on previous data, it is possible that the powerful antioxidant activity of polar extracts is due to the presence of substances with free hydroxyls [23]. In this context, flavonoids possess an ideal structure for the scavenging of free radicals, since they present a number of hydroxyls acting as hydrogen-donators which makes them important antioxidant agents [4,24].

The key role of phenolic compounds as free radical scavengers is emphasized in two important reports [25,26]. Antioxidative properties of essential oils and various extracts from many plants are of great interest in both academia and the food industry, since their possible use as natural additives has emerged from a growing trend to replace synthetic antioxidants by natural ones. Regarding this trend, the study of medicinal plant species has became of great importance, to find and test their bioactive compounds. The results indicate that AE obtained from *Centella asiatica* leaves showed the capacity to donate hydrogen; therefore they present DPPH scavenging activity. This activity might be due to the presence of phenolic and flavonic constituents detected in the samples. The results are in agreement with the recent study, which showed that *Centella asiatica* prevents the oxidative damage existing in several neuropathologies including stroke, Parkinson’s disease and Alzheimer’s disease, improving the antioxidant neurological state related to aging [27].

The cytotoxic activity (IC_50_) of AE against four cancer cell lines and one normal cell line are shown in Table 1. The AE of *Centella asiatica* demonstrated a promising activity against human breast cancer (MDA-MB 231) and mouse melanoma (B_16_F_1_) (648.0 and 698.0 μg/mL, respectivelly), while that for rat glioma (C_6_) the IC_50_ was 1,000.0 μg/mL. On the other hand, the extract was not cytotoxic at the tested concentrations (up to 1,000.0 μg/mL) towards the human lung carcinoma (A_549_) and normal hamster kidney (BHK-21) cell lines.

The cytotoxic activity of AE shown in this work presented in the same range of cytotoxicity (10–1,000 μg/mL) towards the B_16_F_1_, MDA-MB 231 and C_6_ cell lines demonstrated in studies with extracts of other plants with cytotoxic potential in South Korea [5], Tanzania [28], Italy and Jordan [29]. Another report [17] shows that the purified asiatic acid, also present in the AE, decreased the viability of MDA-MB 231 cells in a dose-dependent manner.

Moreover, the extract was not cytotoxic against the A_549_ (lung carcinoma) and BHK-21 (normal kidney cancer) cell lines. These results suggest a possible selectivity of the AE of *Centella asiatica* against some cancer cell lines, as observed for the cisplatin compounds, that are preferentially used for testicular [36] and ovarian cancer [37]. The selectivity of action could be related to the differences in morphology and physiology between tested cell lines, although this is not yet proven. These results are very encouraging, considering that most chemotherapeutic agents found on the market act both on tumor and normal cells [30] and cannot promote a specific treatment for the cancer without causing side effects as a result of damage to normal cells.

Although asiaticoside has not been reported in the scientific literature as a cytotoxic agent, the activity presented here may be related to its major presence in the extract, or even its performance in synergy with other constituents such as phenolic and flavonic constituents identified in this extract. Oxidative stress leads to the damage of membrane lipids, DNA, protein and cellular organelles, contributing to the development of cancer, early-aging, cardiovascular diseases, degenerative and neurological diseases and others. The phenolic constituents, especially the flavonoids, have high antioxidant capacity due to its properties of oxidation-reduction which plays an important role in the adsorption or neutralization of free radicals [4] showing raised biological protection. Since free radicals are involved in the establishment of cancer, the AE containing asiaticoside and other phenolic constituents can act reducing the number of free radicals (antioxidant activity), however at higher concentrations, promotes the cytotoxic effect, as observed in the results of cytotoxic assay.

## 3. Experimental

### 3.1. General

Leaves of *Centella asiatica* were collected in April 2006 from the campus of the Universidade Federal de Juiz de Fora (Minas Gerais, Brazil) and authenticated by Dr Fátima Regina Gonçalves Salimena from the Department de Botany, UFJF, Brazil. A voucher specimen (Nº 24.610) is deposited at the CESJ Herbarium of the Universidade Federal de Juiz de Fora (Minas Gerais, Brazil). Fresh plants were dried and used to prepare the aqueous extract (AE) as previously described [31]. Basically, the extract was prepared by infusion of *Centella asiatica* (200 g) in ultra pure water (4 L) followed by cold maceration for 24 h. The solution was filtered and then freeze-dried to yield a residue that was stored at −80 ºC until use.

A spectrophotometric method explained AOAC [32] was adapted for the phenolic content assay. The AE, was dissolved in methanol to obtain a concentration of 0.5 mg/mL. Folin–Dennis reagent (100 μL) was added to a test tube containing the extracts or fractions (10 μL). Contents were mixed and a saturated sodium carbonate solution (200 μL) was added to the tube. Volume was adjusted to 2 mL by the addition of 1.69 mL of distilled water and the contents were mixed vigorously. Tubes were allowed to stand at room temperature for 25 min and then centrifuged for 5 min at 2,435× g. Absorbance of the supernatant was read at 760 nm. A blank sample of extract was used for background subtraction. Tannic acid was used as standard for the construction of the calibration curve (2–10 μg/mL). The assay was carried out in triplicate.

The procedure used for the quantification of flavonoids is based on the reaction between the flavonoids and aluminum chloride forming a yellow colored complex that can be measured in a spectrophotometer at a wavelength of 420 nm [33]. Rutin was used as standard for the construction of the calibration curve (2–30 μg/mL). For the quantification of the flavonoids content in the AE, an aliquot of 4 mL of chloroform and 6 mL of distillated water was added to 10 mL of the previously obtained sample. The resulting solution was mixed and centrifuged for 3 min at 2,435× g. Two milliliters of the aqueous phase was diluted to 25 mL with 10 mL of the reagent (pyridine, distilled water and aluminum chloride solution 17:80:3, v/v), 12.4 mL of a solution composed of water and dimethyl sulfoxide (1:2, v/v) and 0.6 mL of glacial acetic acid and, soon after vortexing the reaction mixture, the tube was placed in the dark for 15 min and the absorbance was measured at a wavelength 420 nm against the reagent blank. The construction of the calibration curve and the preparation of the sample solutions of the hydroalcoholic extracts for reading were done in triplicate.

The radical scavenging activity was determined by the 2,2-diphenyl-2-picrylhydrazyl hydrate (DPPH) method [34]. The DPPH molecule is a stable-free radical by virtue of the delocalization of the spare electron over the molecule; this delocalization produces a deep violet color, characterized by an absorption band in ethanol or methanol solution centered at about 517 nm. When a solution of DPPH is mixed with that of a substance, that can donate a hydrogen atom, this gives rise to the reduced form (diphenylpicrylhydrazine), with the loss of the violet color. An aliquot (0.5 mL) of ethanol solution containing of the AE obtained from leaves of *Centella asiatica* (0.97–250 μg/mL) was added to 1.5 mL of daily prepared ethanol DPPH solution (0.05 mM). The optical density change at 517 nm was measured 30 min later by a spectrophotometer. A blank was used to remove the influence of the color of the sample. An ethanolic solution of DPPH was used as negative control. Ascorbic acid and butylated hydroxytoluene (BHT) were used as reference drugs, at the same concentrations (0.97–250 μg/mL) as was used for the sample. Results were expressed as mean inhibiting concentration (IC_50_). IC_50_ parameter is defined as the concentration (μg/mL) of substrate that causes 50% loss of DPPH activity (color) and it was calculated by using the following equation: IC_50_ (%) = 100 × (A_0_ – A_s_)/A_0_, where A_0_ and A_s_ are the values for the absorbance of the negative control and the absorbance of the sample, respectively. Tests were carried out in triplicate.

The cytotoxicity assay was evaluated with several tumoral cell lines. To represent more than one embryonic origin cell line, the following cell lines were used in this study: mouse melanoma (B_16_F_1_), human breast cancer (MDA MB-231), rat glioma (C_6_), human lung carcinoma (A_549_) and normal hamster baby kidney line (BHK-21). The MDA-MB 231 and B_16_F_1_ were provided by Dr Ricardo Brentani (Instituto Ludwig de Pesquisa Contra o Câncer, São Paulo, Brazil). The BHK-21 (Baby Hamster Kidney), was provided by CPAFA (Centro Pan-americano de Febre Aftosa, Rio de Janeiro, Brazil). The A_549_ and C_6_ were provided by Dr. Hugo Armelin (Instituto de Química da Universidade de São Paulo, São Paulo, Brazil).

The cytotoxic potential was evaluated using the MTT assay [35]. Cells (10^3^ cells/well) were seeded in RPMI 1640 (Sigma Chemical Co.) medium supplemented with 10% fetal bovine serum (FBS) in 96-well culture plates (Falcon, NJ, USA) and were incubated in a humidified atmosphere with 5% CO_2_, 37 °C, for 24 h until total adhesion to surface. The medium was replaced with fresh supplemented medium containing different concentrations of AE of *Centella asiatica* (0.1–1000 μg/mL). Cells were then incubated at 37°C for 48 h. After this time, the medium was newly refreshed with the same concentration of AE and the plate was incubated again for 72 h. After incubation time, 10 μL of a 5 mg/mL stock solution of MTT in PBS was added to each well containing the cells and incubated again for 4 h. Then, the supernatant without cells was aspirated from each well and 100 μL of DMSO was added to dissolve the dark blue formazan crystals resulting from MTT reduction by homogenization in plate shaker. The extent of MTT reduction to formazan within cells was measured by absorbance at 600 nm using a scanning microplate reader (Stat Fax–2100, Awareness Tecn.). Cultures used as controls did not receive extract.

The percentages of inhibition of cell viability were calculated with the values of viability of cell exposed to the AE and with non-exposed cells, using the *software* GraphPad Prisma 3.0 (GraphPad Software, Inc.). The 50% inhibitory concentrations for cellular population (IC_50_) were calculated by linear regression (SigmaPlot 10.0, Systat Software, Inc.) in the interval of the corresponding concentrations to the curve of MTT metabolization *vs.* log of the used concentrations.

## 4. Conclusions

The results of this study demonstrate the antioxidant capacity of the AE of *Centella asiatica* related to its phenolic and flavonoid constituents and its antitumor potential against cancer cell lines. The results suggest that the potent antioxidant and antitumor activity are justified by the high concentration of phenolic constituents, mainly the flavonoids present in the extract. This study indicates that bioactive molecules present in *Centella asiatica* can be used as a prototype for development of new drugs and/or as a source of antioxidant and antitumor pharmaceutical raw material.

## Figures and Tables

**Table 1.** Antitumor effects of AE of *Centella asiatica* against several cancer cell lines.

	**Cell lines**	**IC_50_ (μg/mL)**	**Taxol^®^ (μg/mL)**
**Tumoral**	B_16_F_1_	698.0	3.55 × 10^−6^
	MDA-MB 231	648.0	6.71 × 10^−6^
	C_6_	1000.0	3.43 × 10^−6^
	A_549_	> 1000.0	6.03 × 10^−6^
**Normal**	BHK-21	> 1000.0	1.06 × 10^2^

N=5; C_6_ (rat glioma), MDA MB-231 (human breast cancer), A_549_ (human lung carcinoma), B_16_F_1_ (mouse melanoma) and BHK-21 (normal hamster kidney fibroblast).
